# Long-term efficacy and safety of sensor-augmented pump for type 1 diabetes

**DOI:** 10.1007/s13340-026-00908-3

**Published:** 2026-06-04

**Authors:** Satoshi Takagi, Junnosuke Miura, Mikako Takita, Shota Mochizuki, Kanako Shimura, Sari Hoshina, Hiroko Takaike, Tetsuya Babazono

**Affiliations:** Division of Diabetology and Metabolism, Department of Internal Medicine, Tokyo Women’s Medical University School of Medicine, 8-1 Kawada-cho, Shinjuku-ku, Tokyo, 162-8666 Japan

**Keywords:** Continuous glucose monitoring, Insulin infusion systems, Sensor-augmented pump, Glycemic control, Type 1 diabetes

## Abstract

**Aims/introduction:**

To evaluate the real-world efficacy of sensor-augmented pump (SAP) for long-term glycemic control and glycemic variability indices.

**Materials and methods:**

This was a single-center, retrospective, observational study that included individuals with type 1 diabetes who switched from multiple daily injections to SAP or continuous subcutaneous insulin infusion (CSII). Changes in HbA1c and body weight up to 3 years were compared between the two groups. Changes in the proportion of participants in each group were also analyzed. In the SAP group, changes in continuous glucose monitoring (CGM) metrics were also examined.

**Results:**

There were 32 individuals in the SAP group and 39 in the CSII group. HbA1c decreased significantly in both groups at 1 year compared with the pre-introduction levels and was maintained for 3 years (− 0.83 ± 0.26%, *p* = 0.009, and − 0.86 ± 0.18%, *p* < 0.001, respectively); however, no significant differences were observed between the two groups after 3 years. Body weight increased significantly in the CSII group after 3 years (2.7 ± 0.9 kg in the SAP group, 2.7 ± 3.3 kg in the CSII group. No significant changes were observed in the CGM metrics.

**Conclusions:**

In individuals who started SAP or CSII, HbA1c decreased after 1 year and was maintained for 3 years.

**Supplementary Information:**

The online version contains supplementary material available at 10.1007/s13340-026-00908-3.

## Introduction

In recent years, remarkable progress has been made in insulin treatment, not only in insulin preparations but also in insulin pumps and continuous glucose monitoring (CGM). In Japan, the first sensor-augmented pump (SAP) was launched in 2015. While pumps used for continuous subcutaneous insulin infusion (CSII) administer a pre-set amount of insulin, SAPs have enhanced functionality through the display and use of CGM data. There are various models of SAPs, such as the predictive low-glucose suspend (PLGS) functional pump (in Japan, launched in 2018), which suspends insulin administration when hypoglycemia is predicted, and the hybrid closed-loop pump (in Japan, launched in 2022), which increases or decreases basal insulin dose based on glucose data. The main insulin therapy options are multiple daily injections (MDI), CSII, and SAP, with MDI being the most common option in Japan. Patient education, including the concept of responsible insulin and how to respond to hypoglycemia, is recommended [1]. Treatment goals are individualized for each patient, but for most patients the goal is HbA1c < 7.0%, which is the goal for prevention of complications [1].

SAP is gaining popularity in Japan. The efficacy of SAP compared to MDI insulin therapy or CSII has been reported to decrease HbA1c without increasing hypoglycemia [2–5]. However, studies comparing the efficacy of SAP with MDI insulin therapy or CSII were short-term observations of 6–12 months [2, 4, 6], and as long as 3 years [5, 7, 8], and reports on long-term glycemic control and weight are limited.

We decided to investigate the real-world, long-term efficacy and safety of SAP therapy. This study aimed to compare long-term changes in glucose control, body weight, and insulin dosage between SAP and CSII users, and to examine changes in glucose indices such as the percentage of CGM active time and CGM metrics such as time in range in the real world.

## Materials and methods

This was a single-center, retrospective, observational study. Participants in this study were individuals with type 1 diabetes treated at the Division of Diabetology and Metabolism, Tokyo Women’s Medical University Hospital aged 18 years or above, who switched from MDI to SAP between February 2015 and December 2019 (SAP group). Individuals who had used SAP before were excluded. As controls, individuals who changed from MDI to CSII (except patch pumps) between April 2014 and December 2019 (CSII group) were included. Type 1 diabetes diagnosis was based on the Japan Diabetes Society diagnostic criteria [9–11]. The decision to choose between SAP or CSII was made after consulting the individual and their physician. The SAP devices available at our institution were: Medtronic Minimed 640G and 620G (Medtronic plc, Dublin, Ireland). The available CSII devices were: Medtronic Paradigms 722 and 712 (Medtronic plc, Dublin, Ireland), TOP Syringe Pump TOP-8200 (TOP Corporation, Tokyo, Japan), and Medtronic Minimed 640G and 620G without CGM. Although the Medtronic Minimed 770G became available during the observation period, participants who started using the hybrid closed-loop function were excluded. Also, LGS and PLGS in MiniMed 640G were also excluded. Participants who started using LGS or PLGS feature were discontinued from observation. For glucose monitoring, individuals in the CSII group underwent self-monitoring of blood glucose (SMBG), and individuals in the SAP group underwent real-time CGM (Enlite sensor). However, because intermittently scanned CGM (isCGM) (FreeStyle Libre, Abbott Laboratories, Chicago, IL, USA) was launched in 2017, some participants in the CSII group used both SMBG and isCGM. Participants were followed up for up to 3 years.

Individuals aged < 18 years were excluded. The observation was terminated when: SGLT2 inhibitors were used; a participant became pregnant; real-time CGM was started in the CSII group; LGS, PLGS, or a hybrid closed-loop insulin pump was initiated in the SAP group; or a participant on CSII was switched to SAP.

### Introduction of insulin pump

In our department, insulin pumps are introduced either on an inpatient or outpatient basis. For the basal insulin dose, the total daily dose (TDD) of insulin for the past week was determined; approximately 80% of the TDD was assumed to be the initial TDD of the insulin pump, and 30–40% of the TDD divided by 24 h was set as the initial value (units/hour) of the basal rate [12]. The amount of bolus insulin was determined based on the amount of rapid insulin at the time of MDI. When carbohydrate counting was performed, the initial insulin sensitivity factor was defined as 1700/TDD (mg/dL/unit), and the initial carbohydrate-to-insulin ratio was defined as 450/TDD (g/unit) [12]. Both values were set with appropriate fine-tuning based on the blood glucose measurements and CGM data. The insulin used was selected in discussion between the physician and the participant. Either aspart, lispro, or glulisine was used.

SAP, CSII, CGM, and SMBG were covered by health insurance. The insulin pump was introduced under the supervision of doctors and nurses at our institution, and follow-up was also performed at our institution. Clinical examinations were performed as part of routine medical care. A typical evaluation schedule at our institution is as follows: blood glucose, HbA1c, and urine analysis are routinely measured every 2 months, and blood count, liver function, and renal function are routinely measured every 2–6 months. For micro vascular complications, retinopathy was routinely evaluated at least annually by an ophthalmologist. Retinopathy was classified according to the Fukuda Classification or Modified Davis Classification. Nephropathy was assessed by measuring urinary albumin/creatinine ratio (uACR), usually once a year, on awakening. An uACR of 30-299 was classified as early nephropathy, and an uACR of 300 or more as overt nephropathy.

Patient education on how to operate the insulin pump, what to do in case of pump issues, and interpretation of CGM results were provided mainly by nurses holding CDEj (an expert in lifestyle guidance in the clinical treatment of diabetes) in the SAP group. The setting of treatment goals and configuration about low glucose suspend were determined individually through consultation between the physician and pump users. For most individuals, the treatment goal was HbA1c < 7% and no severe hypoglycemia.

The primary outcome was a comparison of HbA1c over time and between the SAP and CSII groups over a 3-year period. The secondary outcome consisted of four analyses: (1) a comparison of body weight and insulin use over time and between the SAP and CSII groups over a 3-year period; (2) changes in the proportion of participants in each group who experienced severe hypoglycemia in each year to assess safety; (3) a comparison of the percentage of time that the CGM was active and the CGM metrics among SAP users; and (4) factor analysis of participants who achieved HbA1c < 7%.

HbA1c, body weight, and insulin dosage at outpatient visits were obtained from medical records, and changes in pre-introduction values at 12, 24, and 36 months were determined. The SAP and CSII groups were compared in terms of the percentage of participants achieving HbA1c < 7%, which was the target for most individuals; comparisons were performed annually. Daily insulin dose was divided into total insulin, basal insulin, and bolus insulin, divided by body weight (kg). The insulin change at a given time was calculated as the rate of change calculated from: [(insulin dose at that time − insulin dose before pump introduction)/insulin dose before pump introduction]. Four diabetologists oversaw the outpatient clinic under almost the same policy.

For comparison of severe hypoglycemia frequency, records of severe hypoglycemia were extracted from medical records. The presence or absence of severe hypoglycemia was tabulated 12 months before the start of pumps and every 12 months after the start of pumps, and the presence or absence of severe hypoglycemia during the 12 months before the start of pumps and 25–36 months after treatment. Severe hypoglycemia was defined as “an event requiring another person’s assistance” [13]. Participants introduced to the pump less than 1 year after their first visit to our institution were excluded from this analysis.

For the third analysis, CGM data were obtained for the SAP group only. The CGM metrics were defined as the percentage of glucose data falling within the target range of 70–180 mg/dL as time in range (TIR), the percentage of data above the target range (> 180 mg/dL) as time above range (TAR), and the percentage of data below the target range (< 70 mg/dL) as time below range (TBR) [14]. The percentage of active CGM time was calculated by dividing the number of CGM data points by 288 × number of days. The first month of SAP was excluded from the analysis because CGM may have started a few days after the start of insulin pumps in the outpatient introduction protocol at our institution. The first year was considered 1 month from 1 month after the start of SAP, the second year was considered 1 month from 13 months after the start of SAP, and the third year was considered 1 month from 25 months after the start of SAP to test whether years 2–3 changed compared to the first year. For this analysis, participants whose highest percentage of active CGM time was less than 50% in each year were excluded, because they were considered to have little CGM use.

For the factor analysis of participants who achieved HbA1c < 7%, we investigated factors associated with achieving HbA1c < 7% after 1 or 3 years of treatment for participants whose HbA1c was ≥ 7% before starting the insulin pumps. The following items were tested: treatment group (SAP or CSII), sex, estimated glomerular filtration rate (eGFR), body weight, HbA1c at initiation, total/basal/bolus insulin dose at MDI, age, age of onset, duration of diabetes, presence of severe hypoglycemia in the previous year, and percentage of active CGM time in the first and third years.

### Statistical analysis

Baseline characteristics are presented as mean ± standard deviation or median (interquartile range) for continuous variables. Comparisons between the SAP and CSII groups were made by t-test, and categorical variables were compared by Fisher’s exact test. Changes in continuous variables over time and between-group comparisons were performed using mixed-effects models for repeated measures (MMRM). The model was adjusted for age, eGFR, insulin dose before pump introduction, and retinopathy as fixed covariates. Multiplicity was corrected using the Dunnett–Hsu method with pre-start as a control. Results are shown as least square mean ± SE. The percentage of active CGM time and CGM metrics were calculated using MMRM with the value in the first year as the covariate, corrected by the Dunnett–Hsu method with the value in the first year as the control. McNemar’s test was performed to test for changes in the proportion of individuals experiencing severe hypoglycemia and those who achieved HbA1c < 7%. Statistical analyses were performed using JMP Student Edition 18.2.2 (JMP Statistical Discovery LLC, Cary, NC, USA) and SAS version 9.4 (SAS Institute Inc.).

### Ethics

The study was conducted in accordance with the Declaration of Helsinki. This study was a retrospective observational study, and the requirement for informed consent was waived. Instead, the institution offered participants the opportunity to opt-out. This study was approved by the Ethics Committee of Tokyo Women's Medical University (Date of approval: January 10th, 2023; Approval No. 5535).

## Results

### Baseline characteristics

The baseline characteristics of the participants are shown in Table 1. There were 32 and 39 participants in the SAP and CSII groups, respectively. At baseline, all participants in the SAP group used MiniMed 620G, and all participants in the CSII group used Paradigm 712/722. There were more women than men in both groups. Diabetes duration was significantly longer in the SAP group (25 ± 16 years) than in the CSII group (14 ± 9 years) (*p* < 0.001). eGFR was significantly lower in the SAP group (70.9 ± 29.5) than in the CSII group (103.1 ± 29.2) (*p* < 0.001). HbA1c was 8.1 ± 1.0% and 8.5 ± 1.7% in the SAP and CSII groups, respectively, with no significant difference (*p* = 0.282). There was no significant difference in age (44 ± 12 years in the SAP group, 38 ± 13 years in the CSII group, *p* = 0.071), and body mass index (22.5 ± 3.1 in the SAP group, 22.7 ± 3.0 in the CSII group, *p* = 0.815). Treatment before starting the insulin pumps consisted of MDIs.

**Table 1:** Baseline characteristics

	SAP group (n = 32)	CSII group (n = 39)	*p* value
Male sex, n (%)	8 (25)	9 (23)	1.000^a^
Age (years)	44 ± 12	38 ± 13	0.071
Subtype (fulminant/acute/SPIDDM/not specified)	0/29/1/2	3/33/1/2	0.310^a,b^
Duration of diabetes (years)	25 ± 16	14 ± 9	< 0.001
Body weight (kg)	59.1 ± 12.4	60.2 ± 10.9	0.689
Body mass index (kg/m^2^)	22.5 ± 3.1	22.7 ± 3.0	0.815
eGFR (mL/min/1.73 m^2^)	70.9 ± 29.5	103.1 ± 29.2	< 0.001
HbA1c (%)	8.1 ± 1.0	8.5 ± 1.7	0.282
Total daily dose of insulin (units/kg/day)	0.65 ± 0.21	0.80 ± 0.30	0.022
Daily dose of basal insulin (units/kg/day)	0.25 ± 0.13	0.28 ± 0.10	0.237
Daily dose of bolus insulin (units/kg/day)	0.40 ± 0.15	0.52 ± 0.28	0.039
Retinopathy NDR/SDR/PPDR/PDR/unknown, n (%)	12 (37.5)/4 (12.5)/0 (0.0)/11 (34.4)/5 (15.6)	27 (69.2)/2 (5.1)/0 (0.0)/1 (2.6)/9 (23.1)	< 0.001^c^
Nephropathy Stage 1/2/3/4/5/unknown, n (%)	23 (71.9)/1 (3.1)/1 (3.1)/1 (3.1)/3 (9.4)/3 (9.4)	34 (87.2)/1 (2.6)/0 (0.0)/0 (0.0)/0 (0.0)/4 (10.3)	0.013^c^

*SAP* sensor-augmented pump, *CSII* continuous subcutaneous insulin infusion, *SPIDDM* slowly progressive insulin dependent diabetes mellitus, *eGFR* estimated glomerular filtration rate, *HbA1c* hemoglobin A1c, *NDR* nonproliferative diabetic retinopathy, *SDR* simple diabetic retinopathy, *PPDR* preproliferative diabetic retinopathy, *PDR* proliferative diabetic retinopathy

^a^Fisher’s exact test

^b^For subtypes, participants with unknown subtypes were treated as missing values

^c^Cochran-Armitage trend test

In the CSII group, 21 individuals used isCGM during the observation period (median start time: 28.0 months). All of them continued isCGM until the end of the observation period. The cumulative 3-year continuation rates were 64.0% in the SAP group and 80.4% in the CSII group.

### Changes in clinical indicators and insulin dose

**Fig. 1:**
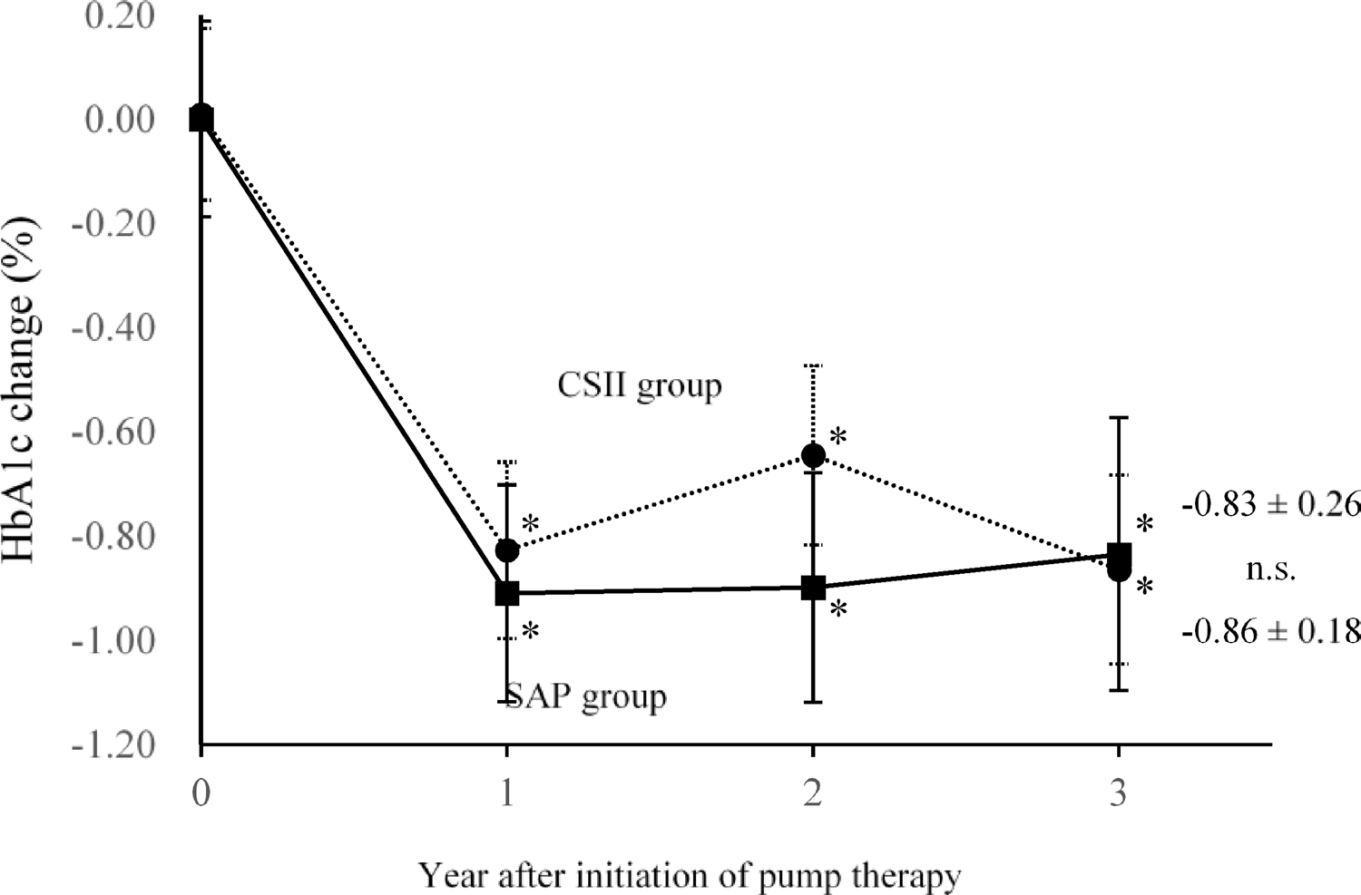
HbA1c change. Changes in HbA1c levels after the initiation of the sensor-augmented pump (SAP group) or continuous subcutaneous insulin infusion (CSII group) are shown. Squares and solid lines indicate the SAP group, and circles and dotted lines indicate the CSII group. Whiskers indicate standard error. *p < 0.05 versus baseline (mixed model for repeated measures). *SAP* sensor-augmented pump, *CSII* continuous subcutaneous insulin infusion, *n.s.* not significant

**Fig. 2:**
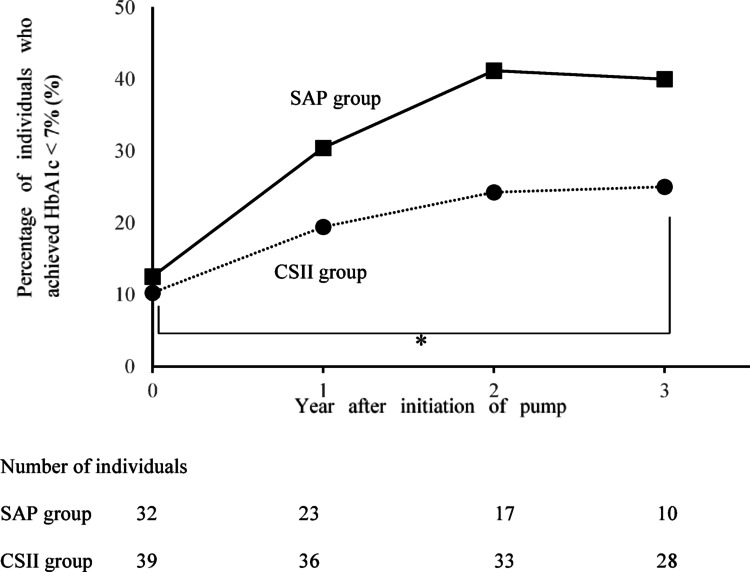
Change in the percentage of participants who achieved HbA1c < 7%. Changes in the percentage of individuals who achieved HbA1c < 7% are shown. Squares and solid lines indicate the SAP group, and circles and dotted lines indicate the CSII group. There was a significant change when compared between the start of the study and 3 years after introduction in the CSII group (McNemar’s test). *SAP* sensor-augmented pump, *CSII* continuous subcutaneous insulin infusion

The changes in HbA1c over time are shown in Fig. 1. Compared to pre-treatment, HbA1c was significantly lower in both groups during the first year, and this was maintained until the third year (after 1 year: SAP − 0.91 ± 0.21, *p* < 0.001, CSII − 0.83 ± 0.17, *p* < 0.001; after 3 years: SAP − 0.83 ± 0.26, *p* = 0.009, CSII − 0.86 ± 0.18, *p* < 0.001). A comparison between the SAP and CSII groups regarding change at 3 years showed no significant difference (*p* = 1.000). Figure 2 shows the change in the percentage of individuals with HbA1c < 7%: 12.5% in the SAP group before introduction, 30.4% at 1 year, 41.2% at 2 years, and 40.0% at 3 years. There was a significant change when compared between the start of the study and 3 years after introduction in the CSII group There was also no significant difference in the proportion of participants with HbA1c < 7% between the SAP and CSII groups at 3 years.

**Fig. 3:**
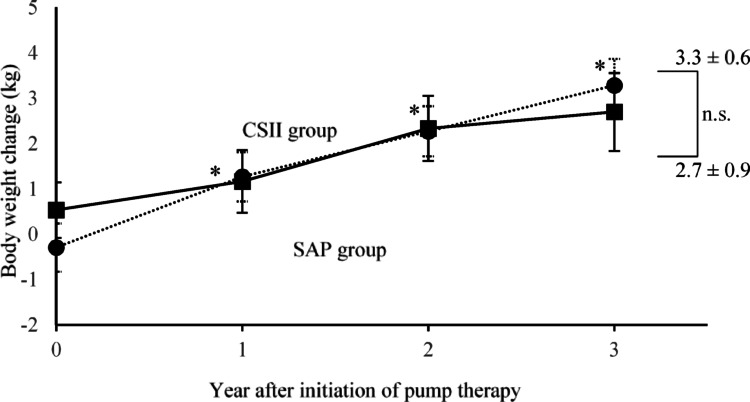
Body weight change. Changes in body weight after the initiation of SAP (SAP group) and CSII (CSII group) are shown. Squares and solid lines indicate the SAP group, and circles and dotted lines indicate the CSII group. Whiskers indicate standard error. *p < 0.05 versus baseline (mixed model for repeated measures). *SAP* sensor-augmented pump, *CSII* continuous subcutaneous insulin infusion

Body weight increased significantly over time in the CSII group but not significantly in the SAP group [SAP group 2.7 ± 0.9 kg (7.1 ± 2.6%), *p* = 0.076, CSII 2.7 ± 3.3 kg (10.6 ± 1.8%), *p* < 0.001 at the third year] (Fig. 3). There was also no significant difference in the 3-year change in body weight between the SAP group and CSII group (*p* = 0.993).

Supplemental Fig. S1 shows the changes in insulin dose (total, basal, and bolus insulin) over time. In both groups, basal insulin tended to increase, and bolus insulin tended to decrease compared with the amount used in the MDI. Changes in basal insulin in the CSII group at 1 and 3 years after initiation of pump therapy and changes in bolus insulin in the SAP group at 2 years after initiation of pump therapy was significant; There were no differences in any of the groups in the amount of change after 3 years.

### Severe hypoglycemia

**Fig. 4:**
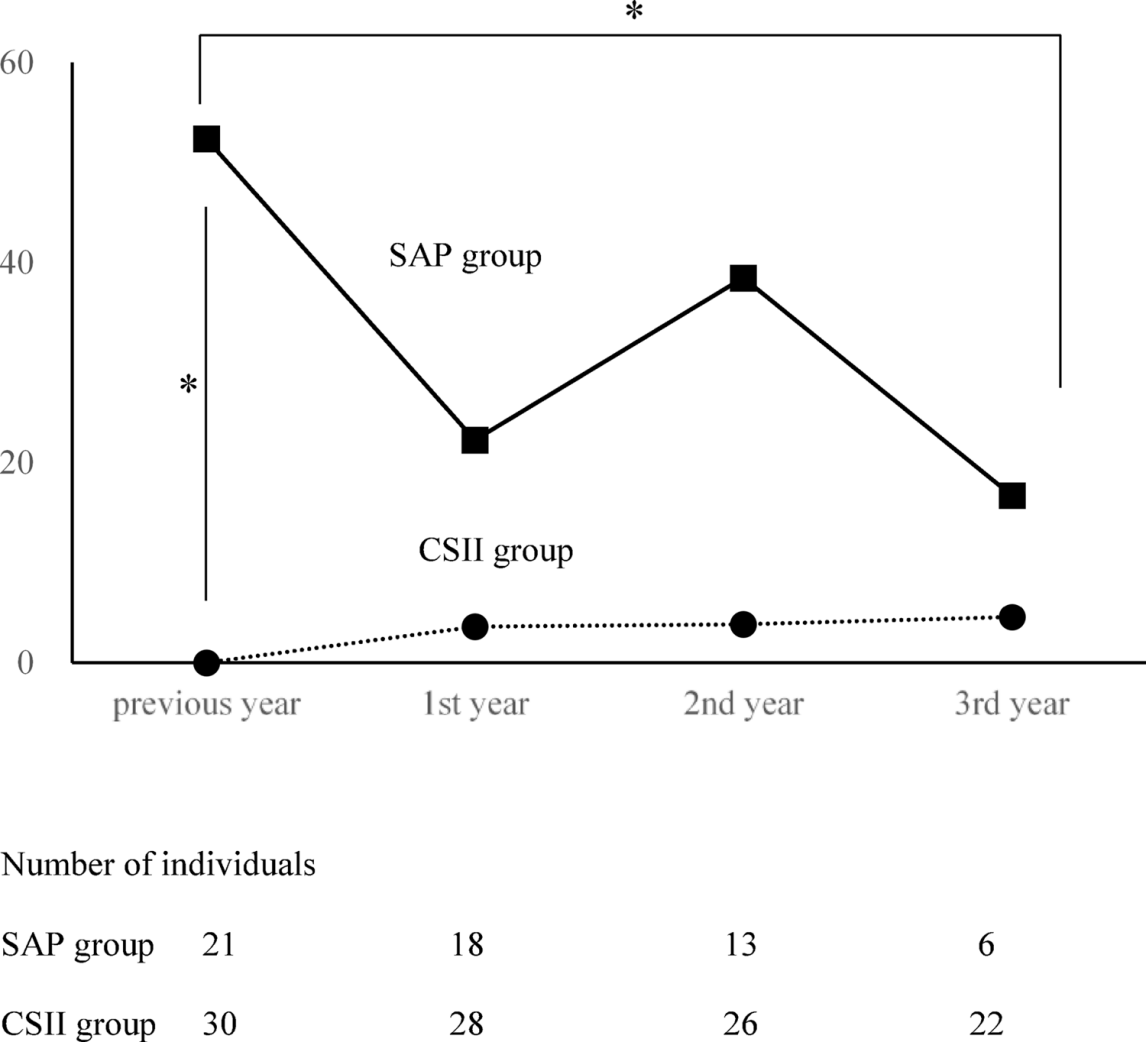
Percentage of individuals who experienced severe hypoglycemia. Percentages of individuals who experienced severe hypoglycemia each year are shown. Squares and solid lines indicate the SAP group, and circles and dotted lines indicate the CSII group. Fifty-two percent in the SAP group and none in the CSII group had severe hypoglycemia before initiation (*p* < 0.001, Fisher’s test). In the SAP group, the proportion of participants experiencing severe hypoglycemia in the third year was significantly lower than that in the previous year (McNemar’s test). *SAP* sensor-augmented pump, *CSII* continuous subcutaneous insulin infusion

Twenty participants (11 SAP, 9 CSII) with < 12 months between their first visit to our institution and insulin pump initiation were excluded from the analysis; 11 participants (52%) in the SAP group and none in the CSII group had severe hypoglycemia before initiation (Fig. 4; *p* < 0.001, Fisher’s test). The proportion of participants experiencing severe hypoglycemia in the CSII group was consistently < 10%. Severe hypoglycemia in the SAP group was slightly lower after introduction than the year before. In the SAP group, the proportion of participants experiencing severe hypoglycemia in the third year was lower than that in the previous year, but not significant (McNemar’s test).

### Percentage of active CGM time and CGM metrics

The percentage of CGM active time was 76.5 ± 3.7% in the first year, with no significant change over time between years 2 and 3 (80.4 ± 3.9 in second year and 77.8 ± 4.1 in third year) (Supplemental Table S1). No significant changes in TIR, TBR, and TAR were observed during the 3-year observation (Supplemental Fig. S2). Additionally, we compared the presence of nephropathy, the presence of retinopathy, and age between TIR over 70% and less than 70% in each year. There were no significant differences in any of above.

### Factor analysis of achieving HbA1c < 7%

Fifty-six participants had HbA1c ≥ 7% before the introduction of pumps. The participants with HbA1c < 7% at 1 year had a lower HbA1c at initiation than those with HbA1c ≥ 7% at 1 year. There was no significant difference in the number of treatment groups (SAP or CSII), sex, eGFR, body weight, HbA1c at initiation, total/basal/bolus insulin dose(units/kg) at MDI, age, age of onset, duration of diabetes, presence of severe hypoglycemia in the previous year, and percentage of active CGM time in the first and third years between the HbA1c < 7% and ≥ 7% groups at 1st and 3rd years, except HbA1c at initiation between the HbA1c < 7% and ≥ 7% groups (at 1st year: 6.3% in the HbA1c < 7% group, 8.5% in the HbA1c ≥ 7% group, *p* < 0.001, analysis of variance; at 3rd year: 7.5% in the HbA1c < 7% group, 8.8% in the HbA1c ≥ 7% group, *p* = 0.024, analysis of variance).

## Discussion

We observed that in individuals who switched from MDI to SAP or CSII for up to 3 years, HbA1c in both groups significantly decreased 1 year after introduction and was maintained until 3 years after introduction. In the CSII group, body weight significantly increased 3 year after introduction. The CGM metrics in the SAP group showed no significant changes.

### Comparison with previous reports

Studies evaluating the long-term outcomes of SAP, including the follow-up study of the SWITCH [Sensing With Insulin pump Therapy to Control HbA(1c)] trial [4, 7], the follow-up study of the Eurythmics trial [15], and the RESCUE (Reimbursement Study of Continuous Glucose Monitoring in Belgium) study [8], have reported that HbA1c in SAP users remained low for 3 years. This is consistent with our study’s report of HbA1c reduction over 3 years after SAP initiation. However, in our study, no group differences in HbA1c reduction from new therapy initiation between the SAP and CSII groups were observed.

As to why no difference was found, first, this was a real-world observational study in which the decision to initiate SAP or CSII was made by the attending doctor and the new user. Second, the characteristics of the two groups differed in that all individuals who had experienced severe hypoglycemia in the year before initiation were initiated on SAP. Therefore, it is possible that fluctuations in blood glucose were larger in the SAP group and that glycemic control was difficult. Finally, 54% of individuals in the CSII group used isCGM, which may be why the difference between CSII and SAP was less pronounced. In other words, although individuals in the SAP group had difficulty managing blood glucose, it was possible to maintain equivalent blood glucose control in the CSII group by making full use of CGM and other measures; however, severe hypoglycemia could not be sufficiently controlled in the SAP group.

As for body weight, the CSII group showed a significant increase from baseline. In the COMISAIR study [6], none of the groups that switched from SMBG to real-time CGM started CSII without real-time CGM or continued MDIs, and SMBG showed significant body weight changes before and after the 52-week observation period. In the STAR 3 study [16], individuals on MDI were randomized to SAP or MDI and observed for 1 year; adult individuals in both groups gained body weight, and no differences between the groups were observed. Total daily dose of insulin did not change significantly in this study. In the RealTrend study [17], daily insulin doses increased after 6 months of observation in both the SAP (MDI replaced by SAP) and the CSII (MDI replaced by CSII) groups, with the increase being significantly greater in the RRT group. The Eurythmics trial [2] showed an increase in the daily insulin dose in the SAP group and a decrease in the MDI group, indicating a difference in treatment between the groups. In contrast, the COMISAIR study [6] showed no significant changes over time in the real-time CGM, SMBG + CSII, or SMBG + MDI groups, and there were no differences among the groups.

### Percentage of active CGM time and CGM metrics

This study found no significant changes in the percentage of active CGM time or CGM metrics.

### Limitations

There are some limitations to this study. First, this was a hospital-based single-center study in a metropolitan area that included a limited number of participants. Therefore, the statistical power of this study is limited, and the findings may not be generalized. Many individuals who wish to receive advanced medical care, including insulin pump therapy and CGM, are referred to our hospital. Therefore, the results of the current study may be biased owing to the inclusion of people who are highly motivated to receive advanced treatment. Therefore, these findings may not necessarily apply to Japanese individuals with type 1 diabetes, including those with newly diagnosed type 1 diabetes.

Second, this was a non-randomized retrospective study, and the choice of treatment was decided through discussions between the doctor and the participants. Therefore, there is a possibility that the SAP group had a large fluctuation in blood glucose levels and was biased towards people with severe hypoglycemia and a strong desire to achieve blood glucose control. Third, the SAP group included PLGS users, and the CSII group included isCGM users; both PLGS and isCGM were expected to improve glycemic control and prevent hypoglycemia, which may have affected the treatment effect in both groups. In the SAP group, CGM data were analyzed at each visit. In the CSII group, CGM data was available only from some of the participants, as they started CGM midway through the study; hence, it was impossible to compare CGM data between the SAP and CSII groups. Fourth, since this was an observational study, CGM data were not available before the introduction of SAP. Additionally, isCGM data in the CSII group were unavailable. Although data from several isCGM users is currently available via cloud, few patients shared their data during the observation period, making detailed data rarely available. Furthermore, the “time in range” data in the medical records could not be used for analysis because the target range was not standardized.

## Conclusions

Individuals with type 1 diabetes who switched from MDI to SAP or CSII were observed for up to 3 years. In both groups, HbA1c was significantly lower after 1 year of introduction than before introduction and persisted through the fourth year. Treatment with an insulin pump may have a better prognosis than MDI because it is expected to lower HbA1c and improve quality of life, such as reducing the frequency of hypoglycemia, over the long term. It is suggested that insulin pump therapy should be proactively selected when suitable.

## Supplementary Information

Below is the link to the electronic supplementary material.

Supplementary Material 1

## Data Availability

Some or all datasets generated during and/or analyzed during the current study are not publicly available but may be available from the corresponding author on reasonable request.
