# Supplementary material for: Long-term efficacy and safety of sensor-augmented pump for type 1 diabetes
Source: Diabetol Int. 2026 Jun 4;17(3):55. doi: 10.1007/s13340-026-00908-3 (PMC13237315; doi:10.1007/s13340-026-00908-3)
Supplement: Supplementary file 1 — Supplementary Material 1 [file 13340_2026_908_MOESM1_ESM.docx]

**Supporting information**

Supplemental Table S1

| Phase | Percentage of CGM active time (%) | p value |
| --- | --- | --- |
| First year (1^st^ month) | 76.5±3.7 |  |
| Second year (13^th^ month) | 80.4±3.9 | 0.094 |
| Third year (25^th^ month) | 77.8±4.1 | 0.823 |

Least square mean ± standard error.

Mixed-effects models for repeated measures (MMRM) with the value in the first year as the covariate, corrected by the Dunnett–Hsu method with the value in the first year as the control.

CGM, continuous glucose monitoring

Supplemental Figure S1. Change in insulin dose

S1a. Total insulin dose

S1b. Basal insulin

S1c. Bolus insulin

a)

Year after initiation of pump therapy

Rate of change (%)

96.6 ± 4.7

n.s.

90.5 ± 6.4

SAP group

CSII group

b)

Year after initiation of pump therapy

Rate of change (%)

117.7 ± 13.5

n.s.

111.4 ± 10.5

SAP group

*

*

CSII group

c)

Year after initiation of pump therapy

Rate of change (%)

93.8 ± 6.5

n.s.

78.3 ± 9.1

SAP group

CSII group

*

## Supplemental Figure S1. Insulin dose change

The rate of change in insulin dose compared to that before initiation (dose for multiple doses of insulin) of SAP (SAP group) or CSII (CSII group) is shown. (a) Total insulin dose. (b) Basal insulin dose. (c) Bolus insulin dose. Squares and solid lines indicate the SAP group, and circles and dotted lines indicate the CSII group. Whiskers indicate standard error. Changes in basal insulin in the CSII group at 1 and 3 years after initiation of pump therapy and changes in bolus insulin in the SAP group at 2 years after initiation of pump therapy was significant.

SAP, sensor-augmented pump; CSII, continuous subcutaneous insulin infusion; n.s., not significant.

Supplemental Figure S2 Change in time in range (TIR), time above range (TAR), and time below range (TBR)

(%)

Time below range

Time in range

Time above range

## Supplemental Figure S2. Change in time in range (TIR), time above range (TAR), and time below range (TBR)

Changes in TIR, TAR, and TBR of SAP group patients in each year are shown. Squares and solid lines indicate TIR, circles and dotted lines indicate TAR, and triangles and dashed lines indicate TBR. The first year considered 1 month from 1 month after the start of SAP, the second year considered 1 month from 13 months after the start of SAP, and the third year was considered 1 month from 25 months after the start of SAP. No significant changes in TIR, TBR, and TAR were observed during the 3-year observation.
